# Stem cell secretome as a mechanism for restoring hair loss due to stress, particularly alopecia areata: narrative review

**DOI:** 10.1186/s12929-022-00863-6

**Published:** 2022-10-05

**Authors:** Ola Salhab, Luna Khayat, Nada Alaaeddine

**Affiliations:** 1 Neuroscience Research Center, Faculty of Medical Sciences, Lebanese University, Beirut, Lebanon; 2 University of Ottawa, Faculty of Science, Ottawa, ON Canada

**Keywords:** Stress, Hair loss, Alopecia areata, Stem cell secretome, Mechanism of restoring hair loss, Hair regeneration, Hair growth, Regenerative therapy/medicine

## Abstract

**Background:**

Living organisms are continuously exposed to multiple internal and external stimuli which may influence their emotional, psychological, and physical behaviors. Stress can modify brain structures, reduces functional memory and results in many diseases such as skin disorders like acne, psoriasis, telogen effluvium, and alopecia areata. In this review, we aim to discuss the effect of secretome on treating alopecia, especially alopecia areata. We will shed the light on the mechanism of action of the secretome in the recovery of hair loss and this by reviewing all reported in vitro and in vivo literature.

**Main body:**

Hair loss has been widely known to be enhanced by stressful events. Alopecia areata is one of the skin disorders which can be highly induced by neurogenic stress especially if the patient has a predisposed genetic background. This condition is an autoimmune disease where stress in this case activates the immune response to attack the body itself leading to hair cycle destruction. The currently available treatments include medicines, laser therapy, phototherapy, and alternative medicine therapies with little or no satisfactory results. Regenerative medicine is a new era in medicine showing promising results in treating many medical conditions including Alopecia. The therapeutic effects of stem cells are due to their paracrine and trophic effects which are due to their secretions (secretome).

**Conclusion:**

Stem cells should be more used as an alternative to conventional  therapies due to their positive outcomes. More clinical trials on humans should be done to maximize the dose needed and type of stem cells that must be used to treat alopecia areata.

## Background

Stem cells are undifferentiated and unspecialized cells of the human body. They are mainly characterized by [1] their ability to self-renew (proliferate rapidly) through symmetrical division, [2] their plasticity because they can differentiate into various types of cells of the organism through asymmetrical division, and [3] their clonality as they derive from the same clone. Stem cells are classified into five major groups depending on their lineage: embryonic stem cells (ESCs), amniotic epithelial cells (AECs), fetal stem cells (FSCs), umbilical cord epithelium (UCE), and adult somatic stem cells (including mesenchymal stem cells (MSCs)). They exist in both embryos and adults [1]. Because stem cells potentially give rise to numerous lineages, and because of their paracrine and trophic effects, they became the center of research as a potential therapies for many diseases therefore they are usually utilized in regenerative and reparative therapy [2].

Stress is any condition that seriously disturbs a person's physiological and psychological balance (homeostasis) [3]. Hippocampus, amygdala, and prefrontal cortex areas are the brain structures primarily involved in the control of the stress response mechanism in autonomic and hypothalamic–pituitary–adrenal (HPA) axis [4]. These regions change both structurally and functionally due to stressful experiences [5]. Structural changes includes neuron replacement, dendritic remodeling, and synapse turnover [6]. Glucocorticoid hormone along with excitatory neurotransmitters such as glutamate alter neuronal architecture leading to dendritic retraction and expansion with modified synaptic density [7]. Indeed, several diseases are caused by stress including increased fat mass osteo-sarcopenia/frailty, cellular dehydration, long-term systemic inflammation [8] and skin related disorders.

Skin is the largest body organ. It is enriched with immune cells, keratinocytes, mast cells, and peripheral nerve endings. Besides, it is a critical element in production of HPA axis components. So, it is considered an active participant in stress response. This leads to various stress-linked skin disorders including Psoriasis, Atopic dermatitis, Vitiligo, Acne, and Alopecia Areata (AA) [9]. An exposure to psychological stress can act as triggering or exacerbating factor for AA [10]. MSCs, in particular, adipose-derived stem cells (ADSCs), are most commonly used in the field of treatment of diseases including skin disorders. Along with their paracrine factors, secretome, MSCs became recently the most promising therapy for stress-induced alopecia. This latter is characterized by immunological disturbances affecting the hair follicle (HF) and contributing to hair loss. MSCs, being able to suppress lymphocyte proliferation and, inhibit complement activation and dendritic cell differentiation from monocytes; were, therefore, considered natural immunosuppressants. Because of this, they are widely used for hair regeneration due to stressful events [2, 11]. There are more than 3000 clinical studies published on Alopecia and around nine studies registered in clinical trials.gov. These studies discuss alopecia and available therapies. On the emergence of cell therapy, and specifically stem cells secretions or secretome as potential treatment for alopecia, three clinical studies were reported, two on clinical trials.gov and one on research gate.net.

In this review, we aim to discuss the role of regenerative medicine, specifically the secretome produced by stem cells, in treating stress-induced alopecia. Besides, we will be shedding the light on the mechanism of secretome action in the recovery of hair loss by reviewing all published in vivo and in vitro studies on stem cells secretions and Alopecia.

## Alopecia and its classification

Alopecia is a disorder in which some or all the hair on the body or head falls off. It might be due to a variety of factors, including stress [12]. It can be classified as scarring and non-scaring ones. Scarring alopecia is considered relatively rare. However non-scarring alopecia includes the following: Alopecia Areata, Anagen effluvium, Androgenic alopecia (most common type), Telogen effluvium, Tinea capitis, Trichorrhexis nodosa and trichotillomania. Other classifications include patchy hair loss or diffuse hair loss or both [13].

### Stress-induced alopecia and the mechanism of action

Alopecia is provoked by a variety of conditions including genetic background and environmental issues. Being genetically predisposed individuals along with enduring stressful life events help the increase of AA [14].

The hair growth cycle consists of three phases: anagen (growth phase), catagen (regression phase) and telogen (resting or inactive phase). During anagen phase, follicles give an entire hair shaft from tip to root. This phase determines the length of hair shaft. In catagen phase, hair shaft differentiation ceases. Each follicle regresses completely in a process including apoptosis of epithelial cells in the bulb and outer root sheath. Hair club is formed, and dermal papilla (DP) remains in contact with epithelium. Following catagen, follicles enter a dormant resting phase (telogen), where no significant proliferation, apoptosis or differentiation is observed [15]. Any disruption in the hair growth cycle will cause hair loss [16].

Experimental studies on animal models primarily mice have tested and proven the tight association between stress/psycho-emotional state and hair loss [17]. Studies on humans still remain limited. Human dermal papilla cells (hDPCs) culture was used to investigate the association between stress and hair loss in humans, and to study the mechanism of hair loss.

Perceived stress (internal or external) can stimulate neuroendocrine immune changes. It was shown that prolonged stress can increase inflammatory cytokines (e.g.INF-ɣ) leading to inflammation and ending up in apoptosis, cell senescence and premature catagen transition. DPCs in hair follicle possess receptors for corticotropin releasing factor (CRF) [18]. When the individual is under stressful events, production of CRF in hypothalamus increases. It binds to its receptors (CRFR1 and CRFR2) on dermal papilla cells inducing high level of cAMP, high level of protein kinase A and consequently high phosphorylation of cAMP response element-binding protein (CREB). This, results in high production of Adrenocorticotrophic hormone (ACTH) which is derived from the prohormone, pro-opiomelanocortin (POMC), by anterior pituitary gland. ACTH will enhance the high production of cortisol, being a main effector in HPA axis, in the adrenal cortex. Presence of massive quantity of cortisol reduces the synthesis and, simultaneously, speeds up the degradation of structural skin components (hyaluronan and proteoglycans) by 40% resulting in dry skin. In addition, these two structural components play a critical role on the normal functioning and cycling mechanism of hair follicle and thus result in disruption of the cycle [16]. Consequently, hair loss is observed.

**Fig. 1:**
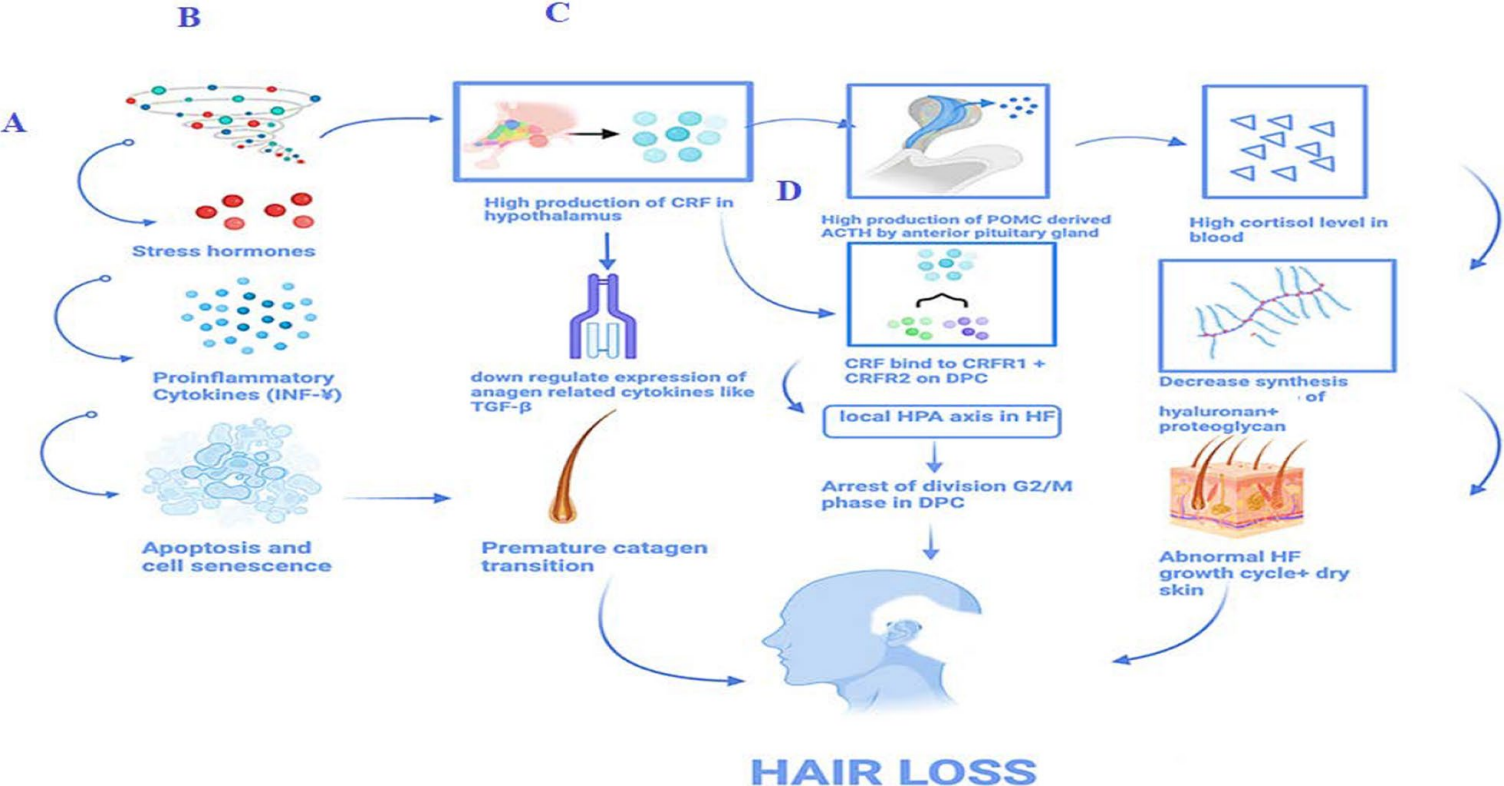
Mechanism of stress-inducing hair loss. **A** Stressful events activate the hypothalamus to produce a high level of CRF which stimulates the anterior pituitary gland to secrete POMC-derived ACTH. The latter contributes to a high level of cortisol in the blood. Cortisol itself decreases the synthesis of hyaluronan and proteoglycan which are responsible for the normal growth of hair shafts. Hence growth cycle is abnormal with dry skin leading to hair loss. **B** External or internal stress through stress hormones causes the release of pro-inflammatory cytokines (INF-γ) which causes apoptosis and cell senescence. This causes premature catagen transition and finally hair loss. **C** High level of CRF is indispensable for less expression of anagen-related cytokines like TGF-β and thus premature catagen transition resulting in hair loss. **D** CRF bind to its receptors CRF1 and CRF2 on DPC creating a local HPA axis in the hair follicle which arrest division in DPC and eventually provoking hair loss

CRF inhibits hair shaft elongation and proliferation of DPCs through arrest of division at G2/M phase. In addition, it accounts for the accumulation of reactive oxygen species (ROS) which also stop the cell cycle. CRF downregulate the expression levels of anagen-related cytokines such as hepatocyte growth factor (HGF), Wnt5a, TGFβ, Vascular endothelial growth factor (VEGF) and versican [18]. Stress resulting from hair loss contributes to a negative feedback elevating hair loss incidence [19]. See Fig. 1.

### Mechanism of stress on alopecia areata

Hair follicle is an immune privilege site, it can tolerate the introduction of foreign antigens without inducing the immune response. Thus, under normal conditions, hair follicle is protected along with its hair follicle stem cells (HFSCs) by this immune privilege. However, its collapse contributes to the pathogenesis of autoimmune hair loss disorders including AA [20]. Indeed, patients with a particular genetic ancestry are susceptible to disorders in hair follicles microenvironment (including trauma, infection and stress), allowing hair follicles self-antigens to be presented to autoreactive CD8+ T cell which will attack and degenerate them. Neurogenic stress is known to affect the immune system. Upon stressful events, CRH factor is released leading to mast cell degranulation, neuropeptide release, high number of CD8+ T cell and Natural killer (NK) cells [21]. CD8+ T cell and NK cells release Interferon ɣ which increases MHC I contributing to collapse of immune privilege [20].

**Fig. 2:**
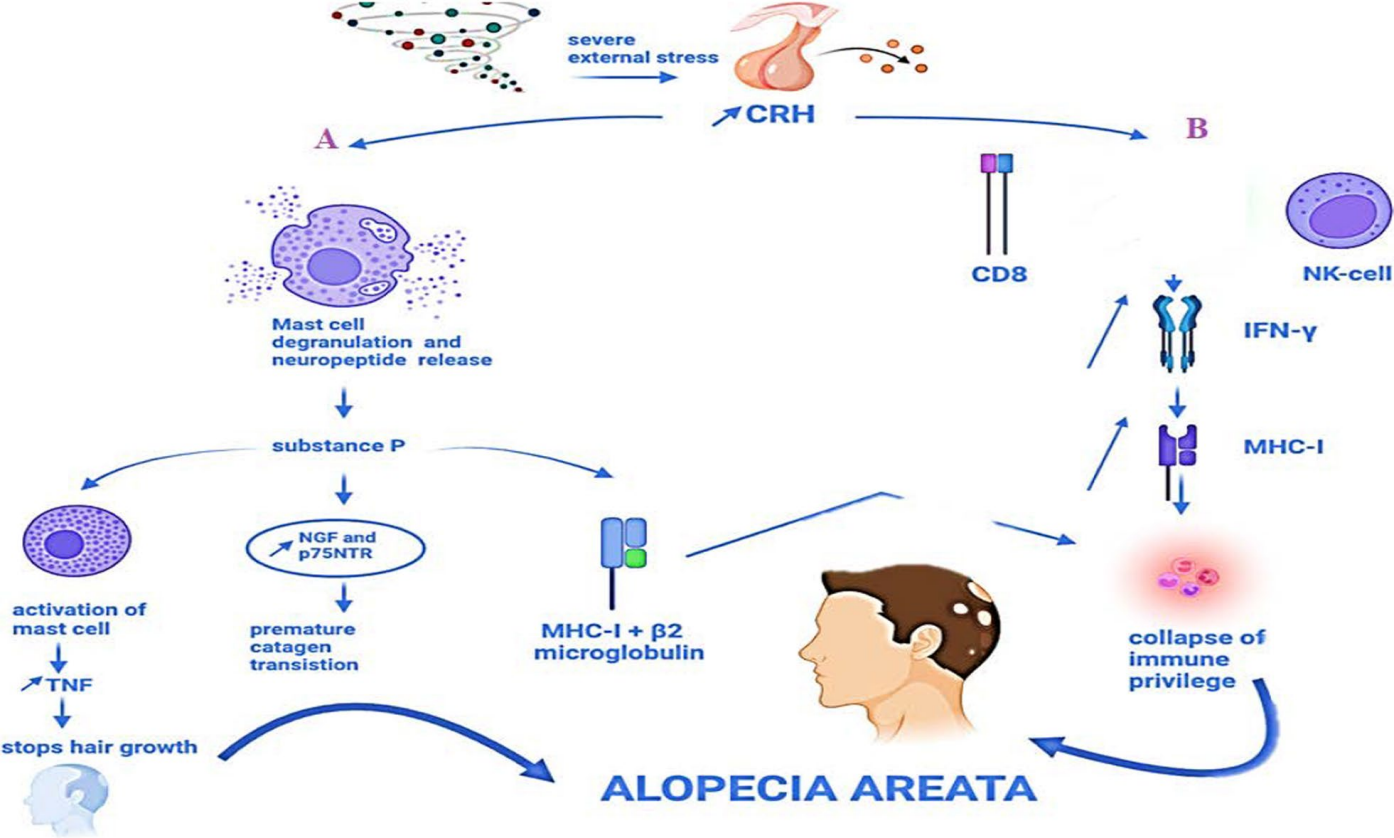
Role of stress in induction of Alopecia Areata through the collapse of immune privilege. **A** CRF released by stress leads to degranulation of mast cells which releases substance P. this substance contributes to the collapse of immune privilege by elevating MHC-I and β2 micro-globulin. Besides, it activates mast cells to release TNF which stops hair growth. It results in premature catagen transition by upregulation of NGF and p75NTR. **B** CRF increases CD 8 + cells and NKs which increases INF-γ and followed by the increase in MHC-I thus collapse of immune privilege and finally AA

Mast cell degranulation and neuropeptides release are responsible for the significant elevation in substance P. The latter increases MHC I and β_2_ micro-globulin and activates perifollicular mast cell and thus enhances neurogenic inflammation. When mast cell is activated, it releases TNF- α which stops hair growth and induces keratinocyte apoptosis. Moreover, substance P stimulates growth factor cascade favoring catagen phase through upregulation of nerve growth factor (NGF) and p75NTR. See Fig. 2

## Available treatments

The patient's age, the location of the hair loss, the degree and severity of the condition, the existence of other medical or psychiatric disorders, and, in certain situations, the findings of a scalp biopsy should be all considered while making treatment recommendations [22].

Many treatments are available to treat alopecia, as shown in Table 1 however only two are FDA-approved: finasteride and minoxidil [23].

**Table 1:** Available treatments for hair loss (Alopecia)

Therapies	Mechanism of action
Dutasteride	inhibition of alpha-reductases
Ketoconazole	androgen receptor blocker
Diphencyprone	antigen competition
Cimetidine, oral prednisolone and flutamide	Antiandrogen
Sulfasalazine	immunosuppressive and immunomodulatory
cyclosporine A	inhibition of T-cell activation
Phototherapy	Immune-modulatory effect due to UV rays
Janus kinase inhibitor	block the T-cell mediated inflammatory response
Surgical transplant therapy	Hair regrowth

Table 1 shows the traditional therapies used to treat hair loss with their mechanism of action in the body. These therapies are not approved by FDA due to their negative side effects.

These therapies have a limited impact and provide unsatisfactory results [24]. With the progress of regenerative medicine, stem cell-based therapies have opened up new avenues to address the issues faced by traditional hair loss treatments. The transplantation of MSCs from adipose, bone marrow, umbilical cord blood and follicles would regenerate hair follicles in the skin as reviewed by Owczarczyk-Saczonek et al. [25]*.* Diffusible factors secreted by stem cells ( such as growth factors and cytokines can activate neighboring cells by paracrine signaling.

The term "stem cell secretome" refers to the soluble factors synthesized by stem cells and utilized for cell–cell communication [26]. It includes proteins, extracellular vesicles (EVs), and nucleic acids, as well as other molecules released into the extracellular space [27]. Secretome accounts for a broad variety of serum proteins, growth factors, angiogenic factors, hormones, cytokines, extracellular matrix proteins, extracellular matrix proteases, and even, in low quantity, lipid mediators and genetic material that are considered to be encoded by around 10% of the human genome [28, 29]. The nutrient medium containing these paracrine molecules along with the stem cells cultured within, is termed “conditioned medium (CM)” [30]. Stem cells produce these substances by both conventional and non-conventional mechanisms, such as protein translocation, exocytosis, and vesicle or exosome encapsulation [31].

MSCs are the most significant stem cells used in regeneration and tissue repair due to their rapid isolation, ex vivo expansion, self -renewal capacity, colony formation, extended population doubling, phenotypic expression pattern, multilineage differentiation potential along with their paracrine trophic effects. After culturing these cells, they secrete secretome and this latter can be taken by syringe after centrifugation and formation of the supernatant. Despite their immune-modulatory, pro-angiogenic, pro-survival, anti-apoptotic, antioxidant, anti-fibrotic, and anti-bacterial properties, some issues come into mind when dealing with stem cells and their secretions [32]. First, the source of MSCs plays an important role in their efficiency. Isolation from different tissues (adult, prenatal and embryonic) shows variations in their plasticity, exosomes, micro-vesicles, mRNA, and mitochondrial transfer abilities. These differences affect the therapeutic outcome of MSC secretome. Second, aging is known to reduce the functional and regenerative capacities of MSCs [33, 34].

MSC administration is considered a feasible and safe procedure with no reported adverse events. Cell source, donor origin, product production, and recipient disease status are important factors related to the safety and efficacy of MSC use. In this regard, the use of bovine proteins in the medium used to culture these cells and the observation of bone tissue formation in animal models, as well as malignant transformation and immune responses, must be evaluated first in order to accept wide clinical applications and registration of this new cell therapy [35].

In regenerative medicine, cell-free therapies have more advantages than classical stem cell-based therapies. In stem cell transplants, the use of secreted molecules might help to prevent immunological compliance, metastatic potential, and infection spreading. As secretome could be produced in substantial quantities ahead of time and made ready for treatment when needed, it might significantly reduce the costs associated with cell lines establishment and maintenance. As a result, this enables them to be used in emergency situations such as infarction and trauma [36].

## In vitro and in vivo studies

### Human umbilical cord blood cells and human dermal papilla cells

IGF-1/Akt/GSK 3β/ β-catenin signaling pathway on h-DPCs is involved in hair regeneration. Intradermal injection of human umbilical cord blood cells (h-UCBs) into C_3_H/HeJ mice, followed by fixation of skin in 4% formaldehyde and addition of Antibody for β catenin were done to monitor β-catenin expression. Results showed high β-catenin protein level which is a positive regulator for hair growth. Protein expression of phosphorylated Akt, phosphorylated GSK3β, β-catenin and proliferating cell nuclear antigen (PCNA) are elevated in h-DPCs and h-UCB-MSCs [37].

Several invitro and in vivo studies were done using secretome mainly derived from ADSCs origin. All studies, as mentioned in Table 2, gave positive results and were able to restore hair loss. These positives outcomes were attributed primarily to the activation of various pathways implicated in hair regeneration such as Wnt/β-catenin signaling pathway. See Table 2.

**Table 2:** Summary of all articles in the literature of stem cells in relation to alopecia

SCs and paracrine factors	Type of study	N	Type of alopecia	Mechanism	Effect	Authors	Refs.
hUCB-MSCs	In-vitro	55	–	VEGF-related β-catenin and p-GSK-3β [SER9] signaling pathway	↑ Viability in DPCs ↑ hair density, thickness, and growth rate	Oh et al	[57]
ASCs	In-vitro (Human DPCs)	–	–	–	Telogen-to-anagen transition Upregulation growth factors	Choi et al	[58]
MSCs	In-vitro	–	Alopecia Areata	Wnt/ β-Catenin pathway Phosphorylation of JAK1 to 3, STAT1, and STAT3	↑ Viability of Human Outer root sheath cells (h-ORSCs)	Lee et al	[59]
Exosomes from ADSCs	In-vitro	–	–	↑ Expression of ALP, versican and α-SMA proteins	↑ DPC proliferation	Nilforoushzadeh et al	[60]
MSC-EVs	In-vivo (C57BL/6 mice) Invitro	17 mice		↑ Bcl-2, phosphorylated Akt and ERK ↑ telogen to anagen ↑expression of wnt3a, wnt5a and versican	↑ DP cell proliferation	Rajendran et al	[61]
ADSC-Exos	In vitro In vivo (C57BL/6 hair-depilated mouse)	15 mice	Immune-Mediated Alopecia	regulating miR-22 Wnt/*β*-catenin signaling pathway TNF-*α* signaling pathway	Hair regrowth	Li et al	[51]
hUCB-MSCs	In-vivo (C3H/HeJ mice) Invitro			Paracrine mechanism	Hair growth	Bak et al	[37]
HF-MSCs	In vitro In vivo: (C3H/HeJ AA)		AA		↓ Hair loss ↓Inflammation around HF	Deng et al	[62]
NSC (TGF-b)	In-vivo (Animal C57BL/6 mice)	20			↑ Keratinocytes and DPCs ↑ Hair shaft length and growth rate	Hwang et al	[63]
ADSC	In-vivo (Animal model- C_3_H/NeH mice)	21			↑ Anagen phase ↑ Hair regeneration, ↑proliferation of hDPCs	Park et al	[64]
ADSCs	In-vivo (Animal model- C57BL/6 J mice)	37		Wnt/β-catenin pathway	↑Hair growth ↑Telogen-to-anagen transition ↑Proliferation, migration and cell cycle progression	Li et al	[65]
ADSCs	In-vivo (Animal model- C57BL/6 J mice)	37		Wnt/β-catenin pathway	↑Hair growth ↑Telogen-to-anagen transition ↑Proliferation, migration, and cell cycle progression	Li et al	[65]
dental pulp stem cells	Invivo (C3H/HeN female mice)	20			↑ Anagen-staged hair follicles ↓ Number of telogen staged hair follicles	Gunawardena et al	[66]
hESCs	In vivo (NUDE mice)				Hair growth	Gnedeva et al	[67]
iPSCs	In-vivo (nude mice)	–	–	–	Unlimited source of folliculogenic cells	Pinto and Terskikh	[68]
ADSC	In-vivo (Human)	1000	–	–	↑ Total number hair shafts	Fukuoka et al	[69]
ADSCs	In-vivo (Human)	22	alopecia	–	↑ Hair number	Fukuoka and Suga	[70]
HF-MSCs (FGF-7)	In-vivo (Human)	21	AGA	ERK activation Wnt signaling pathway	↑ Hair density ↑ Anagen phase	Gentile et al	[71]
HFSCs	In-vivo (Human)	11	AGA	–	↑Hair follicle number and hair density	Gentile et al	[72]
MSCs	In vivo (Human)	4	Alopecia Areata	–	Hair regrowth	Czarnecka et al	[73]
autologous SCs	In vivo (Human)	40	AA and AGA	–	Hair growth	Elmaadawi et al	[74]
stem cell educator therapy	In vivo (Human)	9	AA	–	↑ Hair growth and quality of life	Yanjia Li et al	[75]

Table shows all the in vivo and invitro studies about stem cells and alopecia where all studies yield positive effect. These studies were collected from PubMed database

## Mechanism of action of secretome on treating alopecia

### Wnt signaling pathway and TGF-β signaling pathway

Growth factors (GFs) of stem cell secretome activate DPCs to secrete proteins such as SDF1, MMP3 and biglycan which participate in the induction of Wnt signaling.

**Fig. 3:**
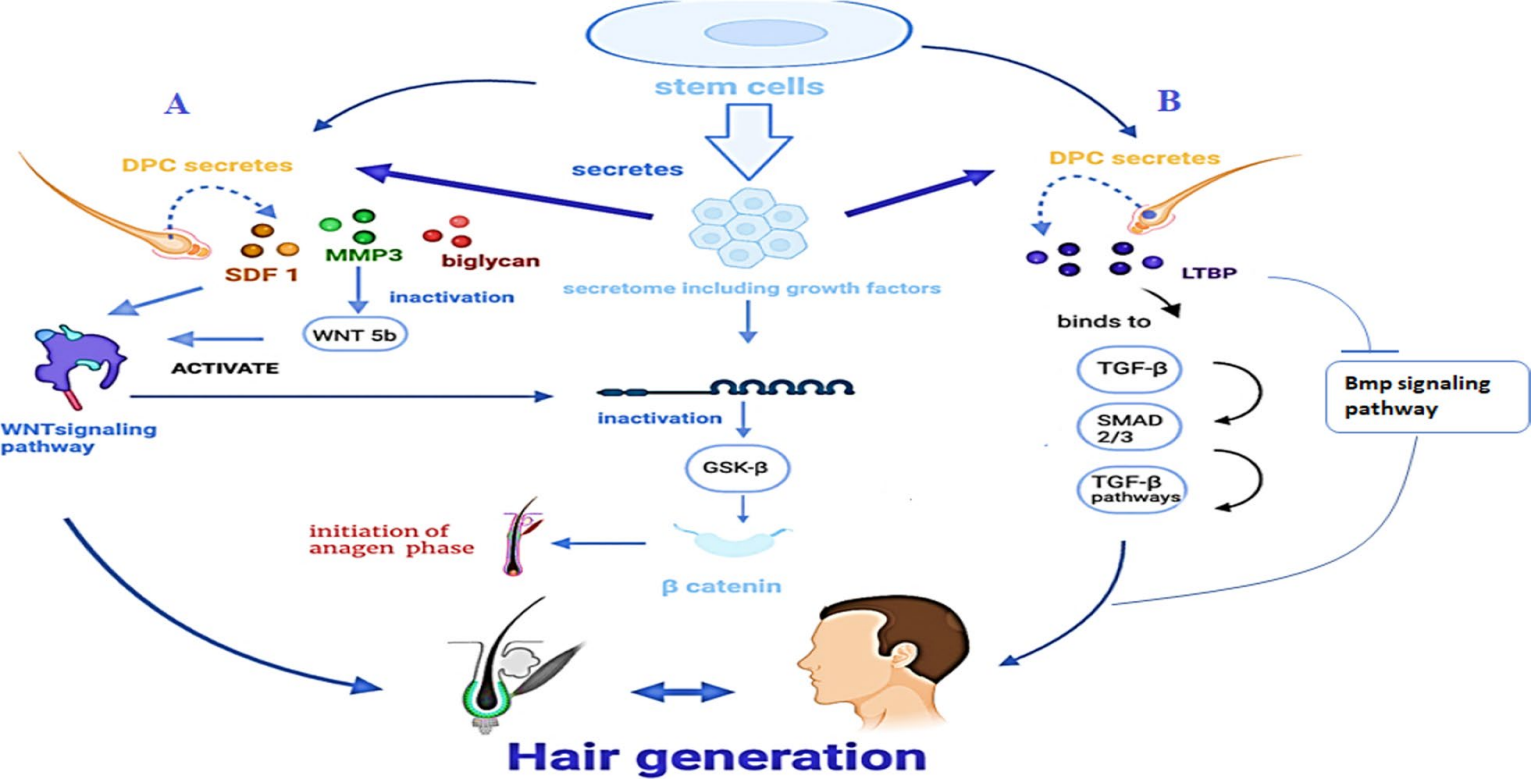
Growth factors in stem cells secretions and their contribution to hair regeneration. **A** Canonical Wnt signaling pathway induction and inactivation of noncanonical Wnt signaling pathway by SDF1, MMP3, and biglycan respectively. The latter proteins are secreted by DPCs due to their activation by GFs. **B** Role of LTBP1 secreted by DPC in hair regeneration. Inactivation of Bmp signaling pathway. Bind with TGF β and activate Smad 2/3 and TGF β pathways

SDF1 and bi-glycan activate Wnt 3a and thus canonical Wnt signaling pathway is induced ending up with high expression level of β-catenin. The latter is a key regulator of hair follicle growth and a primary initiator of anagen phase. At the same time MMP_3_ inhibits the non-canonical Wnt signaling pathway by inactivating Wnt-5b. Wnt-5b is an inhibition factor for β-catenin/Wnt signaling pathway. Other secretory factors of DPCs include LTBP1 which covalently binds to TGF-β and thereby activate the TGF-β signaling pathway. This will lead to the activation of Smad 2/3 pathway in HFSCs and therefore avoids delayed hair regeneration [38]. In addition, LTBP1 participates in BMP signaling pathway inhibition [38, 39]. Inhibition of this pathway is essential since BMP_4_ and BMP_4_ genes inhibit HF development and are associated with maintaining these folicles in the telogen phase [40]. See Fig. 3

### Cytokines and hair regeneration

Secretome consists of VEGF, insulin-like growth factor (IGF), HGF, bone morphogenic proteins (BMPs), interleukin-6 (IL-6), macrophage colony-stimulating factor (M-CSF) and other cytokines. These are highly associated with hair regeneration through various mechanisms [41, 42].

**Fig. 4:**
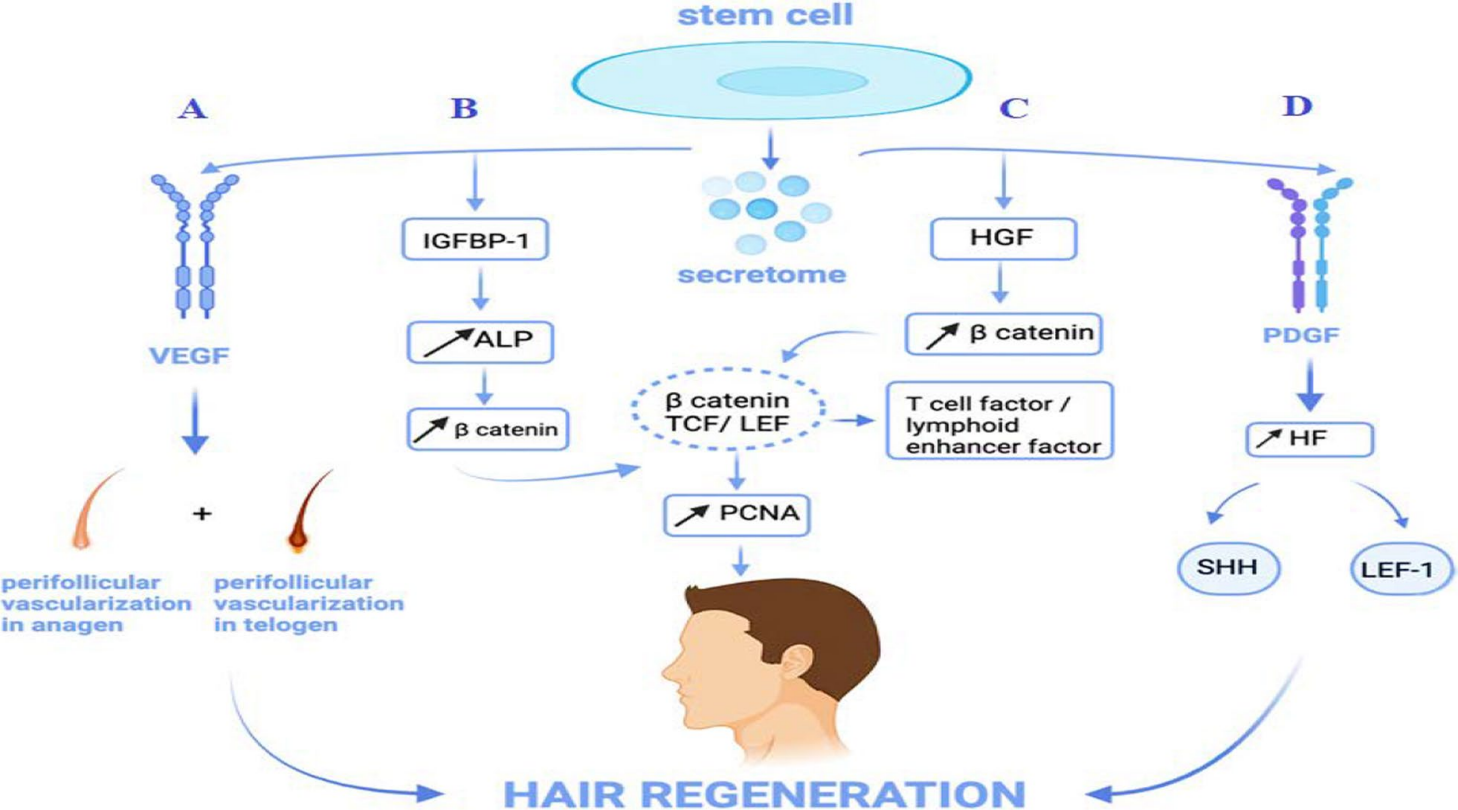
Role of cytokines of stem cell secretions in hair regeneration. **A** VEGF contributes to more perifollicular vascularization in the anagen phase and less in perifollicular vascularization in the telogen phase thus restoring hair. **B**, **C** IGFBP1 and hepatocyte growth factor induce hair regeneration through increasing beta-catenin expression. **D** Role of PDGF in hair regeneration by elevating Shh and LEF1

Starting with VEGF, by promoting perifollicular vascularization in anagen phase and suppressing it in telogen, it speeds up hair regeneration and increases the size of HFs and hair shafts in DPCs. When VEGF secretion is blocked, impaired hair growth is observed [43, 44]. Moreover, both the IGF-1/IGF binding protein-1 complex and BMPs act on DPCs to restore and maintain the potential for hair induction [37, 45]. Indeed, IGF-1 controls hair growth cycle and differentiation of hair shaft. Transgenic mice that express IGF-1 in inner root sheath affects follicular proliferation, tissue remodeling and hair growth cycle as well as follicular differentiation [46]. HGF, another paracrine hormone, may enhance follicular development by boosting-catenin expression [47]. This was manifested by dorsal intradermal injection of 1 µg HGF/SF in 0.1% albumin phosphate buffered saline once daily for 5–7 days in three groups of mice. They showed longer and larger HF in new born mice, retention of anagen HF after 10 days which imply the delay in transition from anagen to catagen [48]. In addition, Platelet derived growth factor (PDGF) is another paracrine factor that induces and maintains anagen phase of murine HFs. PDGF receptors are localized on HFs. It was injected into dorsal skin of C_3_H mice during second telogen phase once daily for 5 days. RT-PCR using extracted RNA from PDGF upregulated expression of HF differentiation related key signaling molecules, sonic hedgehog (Shh), Lef-1 and Wnt-5a [49]. See Fig. 4.

### Exosomes-part of CM- application on hair regrowth

Exosomes are phospholipid bilayer vesicles. They can be produced by various cells including, B cells, T cells, dendritic cells, macrophages, neurons, glial cells, most tumor cell lines, and stem cells. Exosomes may transport valuable cargo between cells in a natural way, facilitating the transfer of genetic material within organisms. Exosome delivery to recipient appears to be a major step in modulating changes in cellular activity as exosomes are crucial for intercellular communication. These vesicles contain a vast range of substances, including proteins, lipids, DNA, and RNA, all of which have regulatory effects on recipient cells [50].

Li et al. investigated the mechanism by which exosomes regenerate hair in a "C57BL/6 hair-depilated mouse model" where they administered, into this model, ADSCs subcutaneously. ADSC-Exosomes increased DPCs proliferation and migration while suppressing apoptosis. Following ADSC-Exosome therapy, RNA-sequencing indicated that the miR-22 and TNF-signaling pathways were significantly downregulated in DPCs. Furthermore, the Wnt/-catenin signaling pathway was activated in the skin of ADSC-Exosome-treated mice, according to qRT-PCR and western blotting data. ADSC-Exosome therapy improved hair regeneration via modifying miR-22, the Wnt/-catenin signaling system, and the TNF-signaling pathway, indicating that ADSC-Exosome may be a potential cell-free therapeutic strategy for immune-mediated alopecia [51].

These bioactive factors stimulate proliferation of DPCs by activation of both Erk and Akt signaling pathways, modulate the cell cycle of DPCs and protect them from damage due to androgens and oxygen species [52, 53].

## Discussion

Alopecia is a topic of great interest. Understanding the pathophysiology and treatment of various alopecias can have a significant impact on a patient's life.

Current and available treatments for this condition have many side effects and show unsatisfactory results. For instance, sulfasalazine has immunomodulatory and immunosuppressive mechanisms that consist of suppression of T cell proliferation and lessening the synthesis of cytokines, particularly interleukin (IL) 6, 1, and 12, tumor necrosis factor alpha, and antibody production. It has been used properly as a long-time period treatment of numerous inflammatory and autoimmune sicknesses, which include inflammatory bowel disorder and rheumatoid arthritis as well as AA. This drug is more effective in patchy alopecia areata than in alopecia totalis/alopecia universalis [54]. Besides, it is able to induce hair regrowth but not capable of altering the course of the disease. Because of their serious effects and inadequate outcomes especially if used for a long period of time, many of the current treatments are not approved by FDA. As a result, therapy using biological factors derived from stem cells are considered to be the best option in such scenarios [24].

Stem-cell based therapies include three distinct prospective mechanisms: transplantation of multipotent stem cells from different sources, application of stem cell-derived secretome and application of stem-cell derived exosomes. These advanced therapies have become recently crucial for treating multiple diseases including alopecia, in particular AA [55].

In this review article, we have revised the mechanism by which stem cell derived factors with their paracrine effect significantly stimulate hair regeneration and reduce inflammation in case of AA. In addition, we have summarized all the articles related to alopecia and stem cells including research on numerous transgenic mice models which have already contributed invaluable knowledge to the field.

One of the most important type of stem cells yielding positive outcomes in hair regeneration is (ADSC)-CM which promotes hair regrowth in a retrospective study where hair density and hair thickness were improved [56]. Won et al. showed that ADSC-CM enhanced the proliferation of DPC-CM and activated Erk and Akt signaling pathways. Akt signaling pathway mediates survival signals whereas Erk signaling pathway plays a role in mitogenesis. In addition, ADSC-CM modulates the cell cycle of DPCs via upregulation of key cell cycle related molecules as cyclin D1 and CDK2 [53]. This is critical since DP size and number of cells correlate with hair growth in anagen phase [56].

Our limitations include collecting information only from English articles and one database which is Pub-med. However its highly important to acknowledge that this review sheds the light on the most advanced and the least harmful therapy that may change the world of medicine and contribute to treatment of multiple debilitating diseases, neurodegeneratives conditions  and  many other  diseases such as cancer [2]. This review may impact the future of regenerative medicine and help clinics treating hair loss to give hope to their patients by using secretome. The secretome from the studies performed by researchers has stopped hair loss and induced hair regeneration. This potential treatment is devoid of serious side effects and is considered a natural non-synthetic product. In our opinion, secretome should be approved as a treatment for many conditions including Alopecia.

## Conclusion

Stress is a psychological condition which leads to several disorders. Chronic stress contributes to hair loss particularly alopecia areata. The autoreactive cells destroy hair follicles leading to hair loss and appearance of alopecia areata symptoms. Stem cells and their secretory factors can be considered as a potential treatment for hair loss and alopecia areata. More trails on humans need to be done in-order to optimize the conditions for this therapy. Number of injections, dose, and time interval should be optimized to reach 100% recovery with no relapse.

## Data Availability

From Pubmed Data base.

## Abbreviations

ADSCs: Adipose derived stem cells
ACTH: Adrenocorticotrophic hormone
AA: Alopecia Areata
AECs: Amniotic epithelial cells
AGA: Androgenic alopecia
AMP: Areata multiples patches
BMP: Bone morphogenic protein
CREB: CAMP response element-binding protein
CM: Conditioned medium
CRF: Corticotropin releasing factor
DPCs: Dermal papilla cells
ESCs: Embryonic stem cells
EVs: Extracellular vesicles
FSCs: Fetal stem cells
GFs: Growth factors
HF: Hair follicle
HFSCs: Hair follicle stem cells
HGF: Hepatocyte growth factor
hDPCs: Human dermal papilla cells
h-UCBs: Human umbilical cord blood cells
HPA: Hypothalamic–pituitary–adrenal
IGF: Insulin-like growth factor
IL-6: Interleukin-6
LTBP1: Latent transforming growth factor beta binding protein
M-CSF: Macrophage colony-stimulating factor
MMP3: Matrix metallopeptidases 3
MSCs: Mesenchymal stem cells
NK: Natural killer
NGF: Nerve growth factor
PDGF: Platelet derived growth factor
POMC: Pro-opiomelanocortin
ROS: Reactive oxygen species
Shh: Sonic hedgehog
SDF1: Stromal cell derived factor-1
UCE: Umbilical cord epithelium
VEGF: Vascular endothelial growth factor
